# Purification of active human vacuolar H^+^-ATPase in native lipid-containing nanodiscs

**DOI:** 10.1016/j.jbc.2021.100964

**Published:** 2021-07-13

**Authors:** Rebecca A. Oot, Yeqi Yao, Morris F. Manolson, Stephan Wilkens

**Affiliations:** 1 Department of Biochemistry and Molecular Biology, SUNY Upstate Medical University, Syracuse, New York, USA; 2 Faculty of Dentistry, University of Toronto, Toronto, Ontario, Canada

**Keywords:** vacuolar H^+^-ATPase, human, membrane protein, protein purification, MS, native lipid nanodisc, ATP6V0a4, ConA, concanamycin A, MSP, membrane scaffold protein, V-ATPase, vacuolar H^+^-ATPase, V_o_, membrane integral proton transport subcomplex, V_1_, cytosolic ATPase subcomplex, VMA21, vacuolar ATPase assembly integral membrane protein 21

## Abstract

Vacuolar H^+^-ATPases (V-ATPases) are large, multisubunit proton pumps that acidify the lumen of organelles in virtually every eukaryotic cell and in specialized acid-secreting animal cells, the enzyme pumps protons into the extracellular space. In higher organisms, most of the subunits are expressed as multiple isoforms, with some enriched in specific compartments or tissues and others expressed ubiquitously. In mammals, subunit *a* is expressed as four isoforms (*a*1-4) that target the enzyme to distinct biological membranes. Mutations in *a* isoforms are known to give rise to tissue-specific disease, and some *a* isoforms are upregulated and mislocalized to the plasma membrane in invasive cancers. However, isoform complexity and low abundance greatly complicate purification of active human V-ATPase, a prerequisite for developing isoform-specific therapeutics. Here, we report the purification of an active human V-ATPase in native lipid nanodiscs from a cell line stably expressing affinity-tagged *a* isoform 4 (*a*4). We find that exogenous expression of this single subunit in HEK293F cells permits assembly of a functional V-ATPase by incorporation of endogenous subunits. The ATPase activity of the preparation is >95% sensitive to concanamycin A, indicating that the lipid nanodisc-reconstituted enzyme is functionally coupled. Moreover, this strategy permits purification of the enzyme’s isolated membrane subcomplex together with biosynthetic assembly factors coiled-coil domain–containing protein 115, transmembrane protein 199, and vacuolar H^+^-ATPase assembly integral membrane protein 21. Our work thus lays the groundwork for biochemical characterization of active human V-ATPase in an *a* subunit isoform-specific manner and establishes a platform for the study of the assembly and regulation of the human holoenzyme.

The internal pH of subcellular compartments, critical to their identity and function, is maintained by a large multisubunit ATP hydrolysis–driven proton pump referred to as vacuolar H^+^-ATPase (V-ATPase). V-ATPase activity is essential for diverse cellular processes including pH and ion homeostasis, endocytosis and exocytosis, protein degradation, zymogen activation, insulin secretion, neurotransmitter release, and pro-growth and developmental signaling pathways including Wnt, Notch, and mTOR (1, 2). The V-ATPase can also be found on the plasma membrane of dedicated acid-secreting cells such as osteoclasts and renal α-intercalated cells where the enzyme is required for bone demineralization and urinary acidification, respectively (3, 4). Aberrant V-ATPase activity is associated with numerous diseases including renal tubular acidosis (5), osteopetrosis (6), neurodegeneration (7, 8), glycosylation disorders (9), and cancer (10). V-ATPase function is also important in both pathogen invasion and host protection, with several pathogenic bacteria found to secrete V-ATPase-specific virulence factors to escape host degradative pathways (11, 12, 13). Furthermore, an increasing number of studies have implicated V-ATPase as being essential for efficient infection by viruses including HIV, influenza, Zika, Dengue, and Ebola, and more recently, the enzyme was identified as being part of the SARS-CoV-2 interactome (14, 15, 16, 17, 18).

The V-ATPase is composed of a cytosolic ATPase subcomplex, called V_1_, and a membrane integral proton channel, termed V_o_ (Fig. 1*A*). V_1_ is composed of eight subunits, A-H, with a stoichiometry of A_3_B_3_CDE_3_FG_3_H, and V_o_ contains six subunits, *a*, *c*, *c*”, *d*, *e*, and *f*, in the ratio *ac*_9_*c*”*def* (19). In mammals, two additional polypeptides called Ac45 or S1 (gene name ATP6AP1) and prorenin receptor (PRR; gene name ATP6AP2) are anchored at the luminal (or extracellular) side of the V_o_ *via* their C-terminal transmembrane α helices (20, 21, 22, 23, 24). V-ATPase is a rotary motor enzyme: cyclic ATP hydrolysis at three catalytic sites in the catalytic hexamer (A_3_B_3_) drives rotation of subunits D, F, *d*, and the membrane integral ring of ten *c* subunits (*c*_*9*_*c”*; *c*-ring) (Fig. 1*A*). During rotation, lipid-exposed glutamic acid residues on the *c* (and *c*”) subunits transfer protons between cytosolic and luminal aqueous half channels located in the membrane integral C-terminal domain of subunit *a* (25). Subunit *a*’s cytosolic N-terminal domain forms a hub for interactions with cytoplasmic subunits (C, H, and three EG heterodimers), and together, these interactions hold the catalytic hexamer static to permit productive rotary catalysis. As a dynamic multisubunit molecular machine, enzyme function requires the concerted action of all ∼30 polypeptides in the complex.

**Figure 1:**
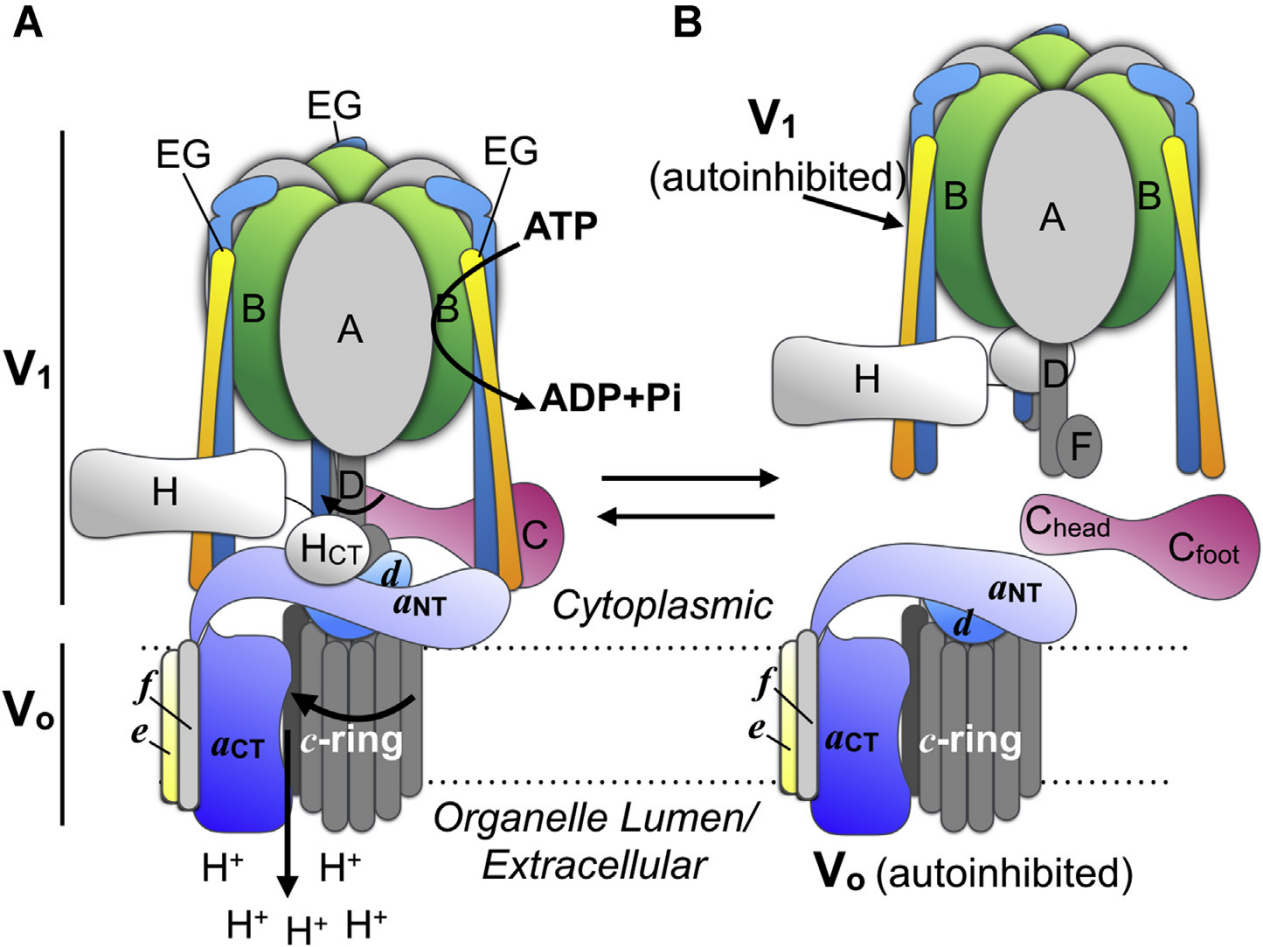
**Subunit architecture and regulation of the human V-ATPase.***A*, schematic of the V-ATPase subunit architecture. Subunits belonging to the cytosolic V_1_-ATPase subcomplex are designated in capital letters (A-H) and those that are part of the integral V_o_ proton channel subcomplex in lower case italics (*a*, *d*, *e*, *f* and *c*-ring). The single copy, ∼100-kDa subunit *a* is shown in *blue* with the C-terminal integral (*a*_CT_) and cytosolic N-terminal (*a*_NT_) domains labeled. For clarity, Ac45 (or S1) and prorenin receptor are not shown. *B*, regulation of V-ATPase activity by reversible disassembly. V-ATPase, vacuolar H^+^-ATPase.

V-ATPase activity is regulated *in vivo* by a unique mechanism termed “reversible disassembly”, characterized by a dramatic (and reversible) structural rearrangement wherein V_1_ is released into the cytosol, leaving behind free V_o_ in the membrane (1, 26) (Fig. 1*B*). Upon disassembly, both subcomplexes become autoinhibited in that V_1_ no longer hydrolyzes MgATP and V_o_ does not catalyze passive proton transport (27, 28, 29, 30). Reversible disassembly has been well characterized in yeast (31, 32) and insects (33, 34), but the process has also been observed in cultured mouse dendritic cells (35, 36), hippocampal neurons (37), rat hepatocytes (38), and established mammalian (39, 40) and human cell lines (15, 41, 42, 43).

A fascinating, yet complicating, aspect in the study of V-ATPase is the presence of subunit isoforms (44, 45). In yeast, only subunit *a* is expressed as two isoforms, presenting an ideal system for study of different isoforms containing V-ATPases (46, 47). Although the two isoforms carry out the same function in the complexes that harbor them, the two enzyme subpopulations differ in subcellular localization, activity, and regulation (48, 49, 50, 51, 52), highlighting the importance of *a* isoforms in shaping unique properties of the enzyme. Furthermore, it has been shown in yeast that the cytosolic N-terminal domain of subunit *a* isoforms is responsible for targeting the enzyme to different biological membranes (48, 50). In higher organisms, most enzyme subunits are expressed as multiple isoforms or splice variants (44, 45), including V_1_ subunits B, C, E, G, H, and V_o_ subunits *d*, *e*, and *a*. Notably, subunit *a* is expressed as four isoforms (*a*1–4) in mammals that, like in yeast, are targeted to different cellular membranes (45). V-ATPase is found on intracellular compartments and organelles in all eukaryotic cells, and ubiquitously expressed isoforms *a*1, *a*2, and *a*3 reside on such membranes (45). Isoform *a*1, while widely expressed, is highly enriched in the brain where it is required for neurotransmitter loading into synaptic vesicles (53, 54). Isoform *a*2 has been observed in early endosomes and Golgi (55, 56) and *a*3 in lysosomes and secretory granules (57, 58, 59). Isoform *a*4 displays the narrowest range of tissue expression, found in the kidney, epididymis, inner ear, and olfactory cells (5, 60, 61, 62). In cell types that are specialized for acid secretion, V-ATPases containing either *a*3 or *a*4 are targeted to the plasma membrane (44). For example, isoform *a*3 is enriched in the plasma membrane of osteoclasts where enzyme function is required for bone resorption (6, 45). In renal α-intercalated cells, isoform *a*4 is enriched in the apical membrane where it acidifies the urine, which is critical to maintenance of systemic pH homeostasis (2, 5). It should be noted that while some tissues or cell types contain an enriched *a* subunit isoform population required for specialized function, such subpopulations coexist with V-ATPases containing different *a* isoforms found on other compartments in the same cell (63, 64). Furthermore, some isoforms of cytosolic V_1_ subunits associate with V_o_ complexes containing specific *a* isoforms, forming unique complexes (Fig. S1) (37, 65, 66). While the isoform composition of some specialized complexes has been experimentally determined or postulated based on expression pattern, a comprehensive picture of the isoforms comprising each V-ATPase in a cell remains unclear. Moreover, it is unknown whether such V-ATPase subpopulations contain any intrinsic functional differences. Despite these layers of complexity, the importance of different *a* isoforms in specialized roles is exemplified by their mutation giving rise to tissue-specific diseases (5, 6, 67, 68). Furthermore, some *a* isoforms are upregulated and mislocalized to the plasma membrane in invasive cancer cells (10, 69), and isoform knockdown or global enzyme inhibition decreases invasion, induces apoptosis, and increases drug sensitivity (10). However, total loss of enzyme function is embryonic lethal (70, 71), and currently available compounds are pan-V-ATPase inhibitors, making the design of isoform-specific therapeutics a priority.

The high level of V-ATPase expression in certain organs or tissues (*e.g.*, brain, kidney, insect midgut, and osteoclasts) has permitted purification of the animal enzyme in sufficient quantities for biochemical and biophysical experiments (72, 73, 74, 75, 76, 77), with much foundational knowledge of the structure and function of V-ATPases coming from such studies. Recently, Wang *et al.* (23) presented high-resolution cryo-EM structures of a human V-ATPase purified from suspension HEK293 cells. While the structures provided a first detailed view of the human *a*1-containing enzyme, the use of a *Legionella pneumophila* virulence factor (SidK; (11)) for affinity capture rendered the enzyme inactive, precluding its use in biochemical studies requiring active enzyme.

The roles of V-ATPase in normal physiology and disease are well documented and continue to be studied in animal models, in case studies of human disease, and on a cellular level. *In vitro* studies of the human enzyme have been more limited, likely due in part to isoform complexity and a lack of a sustainable source of human enzyme for protein purification. Purified, active human V-ATPase of a defined *a* subunit isoform composition would allow analyses of possible intrinsic differences in biochemical properties of enzyme subpopulations, as seen for the yeast enzyme. Furthermore, such a system would greatly simplify characterization of V-ATPase-binding partners and potential regulators, permitting determination of how these interact with the enzyme, whether they are isoform specific, and if they directly impact V-ATPase assembly or function. Moreover, exploring the potential for isoform-specific activity modulation would benefit from *in vitro* biochemical analyses of purified, active enzyme with specific *a* subunit isoform content.

Here, we present purification of active human V-ATPase from suspension HEK293 cells stably transfected with affinity-tagged subunit isoform *a*4. Affinity-captured complexes in native lipid-containing nanodiscs were further purified *via* glycerol density centrifugation, permitting isolation of intact V-ATPase as well as free V_o_ subcomplex. The preparation has a specific ATPase activity of up to 5 μmol × (min × mg)^−1^, which is sensitive to the V-ATPase-specific inhibitor concanamycin A (ConA). MS analysis showed that most subunit isoforms that were part of the purified *a*4-containing complex were of ubiquitous nature. Taken together, the here-developed procedure will allow purification and biochemical characterization of active human V-ATPase complexes with defined subunit *a* isoform content.

## Results

**Figure 2:**
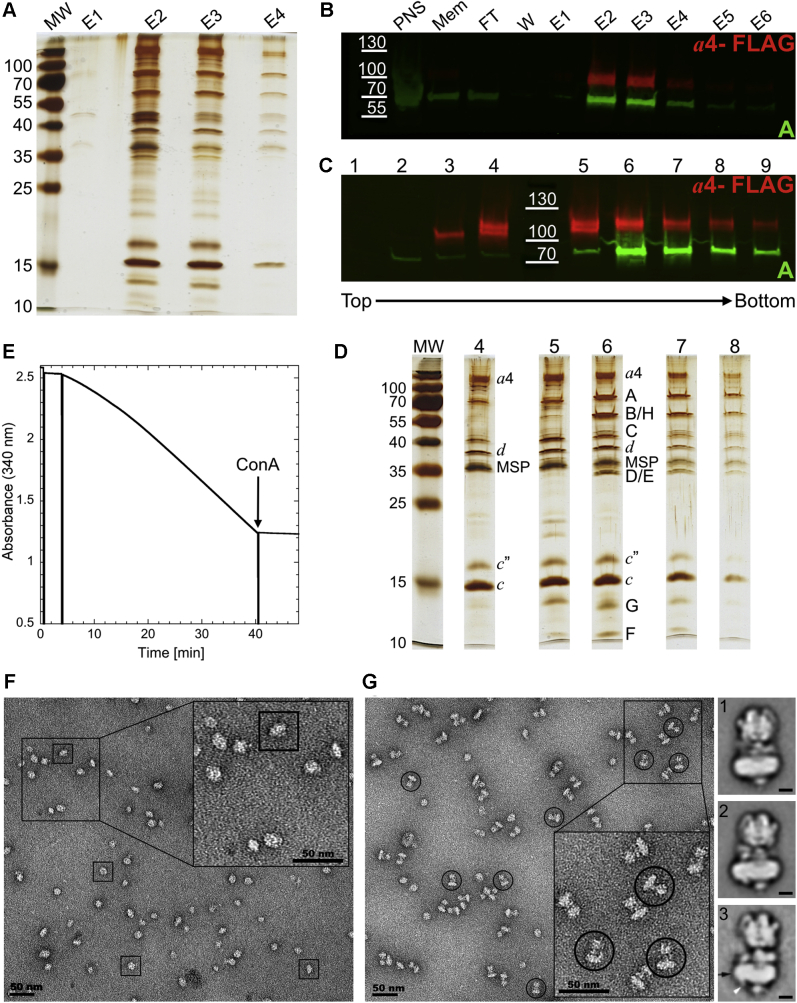
**Purification of the human *a*4-containing V-ATPase.***A*, silver-stained SDS-PAGE showing peak FLAG elution fractions (E1-E4). *B*, Western blot analysis of purification steps (probed for FLAG tag on *a*4 (*red*) and catalytic A subunit, ATP6V1A (*green*)). *C*, Western blot of density gradient fractions probed as in panel *B*. *D*, silver-stained SDS-PAGE of peak gradient fractions. *E*, ATPase assay with 1.3-μg purified V-ATPase. At the indicated time, 200 nM ConA was added to the assay. *F* and *G*, negative-stain electron microscopy of purified V_o_ (*F*) and holo V-ATPase (*G*). Class averages (images 1 and 2) of intact V-ATPase are shown next to panel *G*. For comparison, image 3 shows a two-dimensional average of bovine brain V-ATPase (59). Bar in class averages 1 to 3 is 5 nm. Selections of intact V-ATPases and V_o_ complexes are highlighted by *circles* and *squares*, respectively. E1-E6, FLAG elution fractions; FT, unbound material from the FLAG column; MEM, membrane fraction; PNS, cell lysate after removal of unbroken cells and nuclei; V-ATPase, vacuolar H^+^-ATPase; V_o_, membrane integral proton channel; W, column wash.

So far, mammalian V-ATPase has either been purified from animal organ tissue using conventional biochemical methods or, more recently, *via* affinity capture using the inhibitory virulence factor SidK (22, 23). As there is a need for purified active human V-ATPase of defined isoform composition and because human organ tissue is not widely available, we sought to develop a system for purification of the human enzyme in an *a* subunit isoform–specific fashion. Previously, transient transfection of adherent HEK293 cells with FLAG-tagged *a* isoforms was used to examine subunit glycosylation (78, 79). These studies also showed that the exogenously expressed *a* isoforms were properly posttranslationally modified and assembled with endogenous subunits to form intact V-ATPase complexes (78). We used this plasmid for stable expression of C-terminally FLAG-tagged isoform *a*4 in suspension HEK293 (FreeStyle 293F) cells. This cell line was chosen solely for the purpose of accumulation of sufficient cell mass for purification of the low-abundance V-ATPase and not based on possible endogenous isoform content (evidence suggests that while HEK cells are derived from fetal kidney tissue, the origin of this cell line is most likely adrenal (80, 81)). We find that expression of this single, affinity-tagged, ∼100-kDa *a*4 subunit permits both assembly of intact V-ATPase (by incorporation of endogenous subunits) and affinity purification of the resulting ∼1-MDa multisubunit complex. In our hands, initial preparations of the enzyme purified in detergent displayed very little activity (Fig. S2), in accord with early biochemical purifications of the animal enzyme that reported a lipid requirement for activity (73, 82, 83). Lipid nanodiscs, synthetic lipid bilayers stabilized by binding membrane scaffold protein (MSP) (84), have been widely used for structural and biochemical analysis of membrane proteins. Previously, our laboratory has found that reconstitution of yeast V-ATPase into native lipid-containing nanodiscs yielded robust specific activity and superior stability of the complex (85). Here, a similar procedure was used for purification of the human enzyme, wherein a dense membrane/organelle containing fraction was solubilized in dodecyl maltoside in presence of MSP, followed by slow detergent removal, and spontaneous formation of lipid nanodiscs. Nanodisc-reconstituted total membrane proteins were then applied to a FLAG column for affinity capture of the *a*4-containing V-ATPase (Fig. 2*A*). As the V-ATPase is regulated *in vivo* by reversible disassembly of the V_1_ and V_o_ subcomplexes (Fig. 1*B*), the assembly state of the complex is commonly assessed by probing for presence of subunits from both V_1_ and V_o_ subcomplexes. Western blot analysis of the fractions eluted from the FLAG column using antibodies against both *a*4-FLAG (V_o_) and the catalytic A subunit (V_1_) revealed the presence of intact V-ATPase (Fig. 2*B*). The peak fractions were then concentrated and applied to a glycerol density gradient, which revealed two overlapping peaks, one containing isolated V_o_, and the other, intact holo V-ATPase (Fig. 2*C*). Silver-stained SDS-PAGE of gradient fractions confirms the presence of all major enzyme subunits for V-ATPase and V_o_ complexes (Fig. 2*D*). The average yield from 20 g of cells (∼1.6 l culture) of purified, nanodisc-reconstituted V_o_ and intact V-ATPase is ∼100 μg and 135 μg, respectively. Moreover, fractions containing the intact enzyme display a specific activity of ∼2 to 5 μmol × (min × mg)^−1^ (3.7 ± 0.9 (s.e.m) μmol × (min × mg)^−1^ for three independent preparations) that is inhibited >95% by the specific V-ATPase inhibitor ConA (Fig. 2*E*). ConA inhibits V-ATPase by binding to the *c*-ring in V_o_; therefore, ATP hydrolysis (on V_1_) that is inhibited by ConA indicates that the enzyme is properly assembled and functionally coupled (82, 86). Analysis of the gradient fractions containing isolated V_o_ and intact V-ATPase (Fig. 2, *F* and *G*) by negative-stain electron microscopy reveals that the samples are monodisperse with the characteristic morphology for the two complexes.

**Figure 3:**
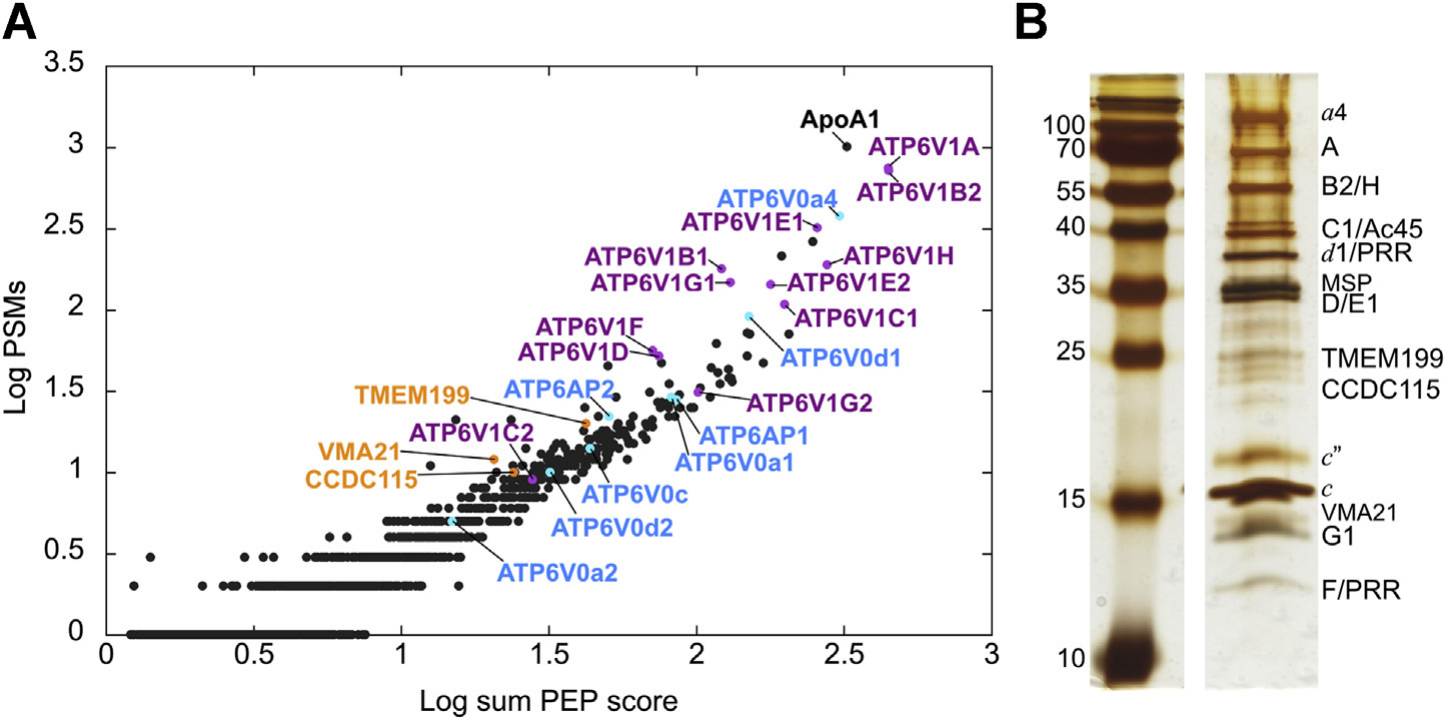
**MS analysis of the purified *a*4-containing human V-ATPase.***A*, plot showing proteins identified by MS in gradient fraction 6 (see Fig. 2, *C* and *D*), labeled with gene names of V-ATPase subunits and accessory proteins (V_1_, *purple*; V_o_, *blue*), as well as assembly factors (*orange*). ApoA1 corresponds to the membrane scaffold protein (MSP) from the lipid nanodiscs. PEP score = sum of the negative log of PEP values for associated PSMs (Proteome Discoverer). *B*, silver-stained SDS-PAGE gel labeled with subunit isoforms, accessory proteins, and assembly factors identified by MS analysis of excised bands (Figs. S4 and S5). The *c*-ring subunits (*c*, *c”*) were annotated based on their characteristic staining patterns. PEP, posterior error probability; PSMs, peptide spectrum matches; V-ATPase, vacuolar H^+^-ATPase; V_o_, membrane integral proton channel; V_1_, cytosolic ATPase subcomplex.

In humans, although a core set of subunits are invariant (A, D, F, *f* and *c*-ring), many subunits (B, C, E, G, H, *a*, *d*, *e*) are expressed as multiple isoforms or as splice variants with some tissue enriched and others ubiquitously expressed (44, 45). As expression of isoform *a*4 is enriched in the kidney along with V_1_ subunit isoforms B1, C2, E1, and G3 and V_o_ isoform *d*2 (5, 87, 88), it was of interest to analyze which subunit isoforms co-purified with exogenously expressed *a*4. MS analysis revealed the presence of largely ubiquitous subunit isoforms (B2, C1, E1, G1, and *d*1) copurifying with isoform *a*4 (Fig. 3, *A* and *B*, Figs. S3–S5, Supplemental Data 1). However, lower scoring hits included tissue-specific isoforms (B1, C2 (kidney), E2 (testes), G2 (brain)) (44, 87, 88, 89) and may indicate the presence of minor populations or “hybrid” complexes containing multiple isoforms of multicopy subunits, as had been suggested previously for V-ATPase immunoprecipitated from murine kidney (66). Our analysis indicates that when exogenously expressed in HEK cells, *a*4 forms a fully functional complex containing isoforms B2, C1, G1, E1, and *d*1, likely to be the more abundant ones present in these cells. Therefore, this purified complex differs from the major kidney V-ATPase in V_1_ subunit isoforms B, C, and G. Interestingly, it was previously found that despite the enrichment of *d*2 expression in the kidney, isoform *d*1 is part of the renal V-ATPase (66) and as such the composition (*a*4, *d*1) of the V_o_ complex presented here contains the same isoform composition as that found enriched in renal cells. While containing some differences in the isoform content, the specific activity of the purified complex presented here is similar to that previously reported for bovine kidney V-ATPase (73).

MS analysis of gradient fractions also revealed the presence of factors required for biosynthetic assembly of the V_o_ complex (Fig. 3, *A* and *B* and Figs. S3–S5, Supplemental Data 1), with the denser of the two peak V_o_-containing fractions enriched in assembly factors (Fig. S6). Biosynthetic assembly of the V_o_ complex occurs in the endoplasmic reticulum, and although well characterized in yeast (90), less is known about the process in mammals. Recently, mammalian orthologs to yeast assembly factors Vma22p, Vma12p, and Vma21p (91, 92, 93) have been identified as coiled-coil domain–containing protein 115, transmembrane protein 199, and vacuolar H^+^-ATPase assembly integral membrane protein 21, respectively (94, 95, 96). Our analysis indicates that along with intact V-ATPase, and V_o_, purification from organelle membranes using tagged isoform *a*4 permitted isolation of assembly intermediates from the endoplasmic reticulum membrane. Furthermore, overexpression of *a*4 may lead to some saturation of the assembly pathway, permitting capture of what would otherwise be transient assembly intermediates and providing exciting new opportunities for the study of enzyme assembly in humans.

## Discussion

Here, we report purification of lipid nanodisc reconstituted human V-ATPase with defined *a* subunit isoform composition. Stably expressed isoform *a*4 associates with ubiquitous isoforms of other enzyme subunits to form an active complex. The preparation is monodisperse and displays robust specific activity that is >95% ConA sensitive. The yield of purified holo V-ATPase is ∼85 μg per liter of culture, approximately eight times higher than that reported for affinity capture of endogenous human enzyme using SidK (97), highlighting the utility and efficiency of moderate and stable overexpression of isoforms for purification. Our procedure provides a means for purification of active human V-ATPase that is suitable for biochemical and structural studies. Next to holo V-ATPase, the protocol also allowed purification of isolated V_o_ complexes. Free autoinhibited V_o_, which can be generated as a result of V-ATPase regulation by reversible disassembly *in vivo*, is stabilized by a conformational change in subunit *a* (25, 98). In yeast, enzymes containing different *a* isoforms have different propensities to undergo this mode of regulation (48, 51). In mammals, V-ATPases containing isoform *a*1 (enriched in the brain) reversibly disassemble (37), and autoinhibited V_o_ has been purified and characterized from bovine brain clathrin-coated vesicles (27, 98). However, it remains unclear whether reversible disassembly in mammals is restricted to specific isoforms, as in yeast. Previously, we have presented a defined *in vitro* system for analysis of reversible disassembly of the yeast V-ATPase using purified V_1_ and V_o_ subcomplexes (99). Establishment of such a system for the human enzyme will benefit from purified human V_o_ subcomplexes of defined isoform composition. Such studies will further our understanding about this mode of regulation in humans, information that will be required as reversible disassembly has been proposed as a possible target for therapeutic modulation of enzyme function (100). Furthermore, although much recent progress has been made on the structure of the mammalian enzyme (22, 24), including from humans (23), there is currently no high-resolution information for the isolated V_o_ complex from mammals. Moreover, we have isolated a population of V_o_ bound to assembly factors, likely constituting assembly intermediates. Mutations in assembly factors coiled-coil domain–containing protein 115, transmembrane protein 199, and vacuolar ATPase assembly integral membrane protein 21 (VMA21) cause abnormal protein glycosylation disorders and liver disease (94, 95, 101), and as such, a more thorough characterization of the assembly process in mammals is needed. The purification of such complexes will provide a valuable tool for furthering our understanding of the V-ATPase assembly process in humans.

Taken together, the approach presented here provides a new tool and a platform for addressing questions related to enzyme function, biochemical properties, regulation, and biosynthetic assembly, in an isoform-specific way. This strategy can, in principle, be extended to expression of other *a* isoforms, thereby shedding light on whether various isoform-containing V-ATPases display inherently different specific biochemical properties as observed in yeast. Purified, active human V-ATPase will simplify the analysis of potential V-ATPase regulators and binding partners, allowing examination of their direct effects on enzyme activity and/or assembly. Importantly, as specific *a* isoforms are upregulated and mislocalized in cancer cells, purified, active human enzyme of defined *a* isoform composition will have the potential to greatly assist in the selection, development, and characterization of isoform-targeted therapeutics.

## Experimental procedures

A detailed description of human cell culture and biochemical experiments and a table containing information for key resources can be found in SI Methods.

### Expression and purification of MSP

MSP1E3D1 carrying N-terminal 7× His and Avi tags separated by a PreScission protease cleavage site was expressed and purified as previously described (99), with modifications detailed in SI Methods. Briefly, MSP was expressed in *Escherichia coli* BL21(DE3) and purified using Ni-NTA affinity chromatography. MSP was dialyzed against low-ionic strength buffer, lyophilized, and stored at −80 °C until use.

### Isoform a4-2× -FLAG stable expression and cell growth

Suspension HEK cells (293F; FreeStyle cells) were maintained in FreeStyle media in vented shake flasks at 37 °C in a humidified 8% CO_2_ atmosphere. Cells were transfected with a pcDNA3.1 vector containing the coding sequence for isoform *a*4 (ATP6V0a4) carrying a C-terminal 2× FLAG tag (78) and selected for using Geneticin. For protein purification, 1.6-l batches of cells were harvested by centrifugation, flash-frozen in liquid nitrogen, and stored at −80 °C until use.

### Purification of human V-ATPase in lipid nanodiscs

A detailed method for purification of the human V-ATPase can be found in SI Methods. Briefly, cells (∼20 g) were lysed in a Dounce homogenizer, and unbroken cells and nuclei were removed by low-speed centrifugation. A dense membrane/organelle fraction was collected by centrifugation (14,600*g*), resuspended at ∼16 mg/ml membrane protein in ∼15 mg/ml MSP, and extracted in 1% dodecyl maltoside. After detergent removal with 0.4 g/ml Bio-Beads, nanodisc-reconstituted membrane protein was mixed with 1.5 ml of αFLAG resin for affinity capture of FLAG-tagged *a*4-containing complexes. Elution fractions were pooled, concentrated, and applied to an 11-ml (20–50%) glycerol density gradient. Fractions were collected from the top of the gradient and analyzed by Western blot and silver-stain SDS-PAGE, and protein concentration was determined using a modified Pierce BCA assay.

### Immunoblot

Samples were separated on gradient SDS-PAGE (4–20% acrylamide) gels, transferred to low-fluorescence polyvinylidene difluoride membranes, and probed for the catalytic A subunit (ATP6V1A) and the FLAG tag on *a*4. Details can be found in SI Methods. Antibody source information is listed in the Key Resources Table.

### ATPase activity measurements

ConA-sensitive ATPase activities were measured at 37 °C using a coupled enzyme assay as previously described (29, 99).

### Negative-stain electron microscopy

Samples were spotted on carbon-coated copper grids and stained with 1% uranyl acetate. Grids were examined in a JEM-1400 transmission electron microscope (JEOL) operating at 80 kV. Micrographs were recorded with an Orius SC1000 (model 832) CCD camera (Gatan Inc) at 200,000× magnification. Two-dimensional averages were generated as described in SI Methods. Briefly, a dataset of ∼15,400 particles selected from 40 micrographs was processed as described (75).

### MS sample preparation and analyses

In-gel trypsin digestion was carried out on excised gel bands from two independent purifications. In-solution digestion of peak gradient fractions was carried out using filter-assisted sample preparation (102) and peptides desalted using mixed-mode cation exchange stage tips (103). For LC-MS/MS, samples were dissolved in water containing 2% acetonitrile and 0.5% formic acid. Samples (0.5 μg) were injected onto a nano-LC (C18) column connected inline to an Orbitrap Lumos mass spectrometer *via* a nanoelectrospray source operating at 2.2 kV. The MS data were searched using Sequest HT in Proteome Discoverer (version 2.4, Thermo Scientific) against the human proteome in UniProt. Label-free quantification was performed in Proteome Discoverer. Details of the MS sample preparation and analyses can be found in SI Methods.

## Data availability

All data presented are contained within the article and supporting information. Raw MS data were deposited into the Figshare data repository under 10.6084/m9.figshare.14589453.

## Supporting information

This article contains supporting information (22, 24, 44, 45, 66, 75, 78, 85, 89, 99).

## Conflict of interest

The authors declare that they have no conflicts of interest with the contents of this article.
