# Supplementary material for: Purification of active human vacuolar H+-ATPase in native lipid-containing nanodiscs
Source: J Biol Chem. 2021 Jul 13;297(2):100964. doi: 10.1016/j.jbc.2021.100964 (PMC8353480; doi:10.1016/j.jbc.2021.100964)
Supplement: Supplementary Methods, Table S1 and Figures S1–S6 [file mmc2.pdf]

Purification of active human vacuolar ATPase in native lipid-containing nanodiscs

Rebecca A. Oot[1], Yeqi Yao[2], Morris F. Manolson[2], Stephan Wilkens[1]

[1]Department of Biochemistry and Molecular Biology, SUNY Upstate Medical University, Syracuse, NY 13210, USA

[2]Faculty of Dentistry, University of Toronto, Toronto, Ontario M5G 1G6, Canada

Supporting Information

Supplementary Methods

Supplementary Table S1: Key resources

Supplementary Figure S1: V-ATPase isoforms and specialized complexes

Supplementary Figure S2: ATPase activity of detergent solubilized *a*4-containing V-ATPase

Supplementary Figure S3: Solution mass spectrometry analysis of additional gradient fractions

Supplementary Figure S4: Mass spectrometry identification of proteins excised from SDS-PAGE

Supplementary Figure S5: Mass spectrometry identification of proteins excised from silver stained SDS-PAGE

Supplementary Figure S6: Mass spectrometry label-free quantification of gradient fractions

Supplementary Spreadsheet: Excel spreadsheet showing mass spectrometry data for V-ATPase subunits, isoforms, and assembly factors identified in gradient fractions

Supplementary Methods

Expression and purification of membrane scaffold protein (MSP)

MSP1E3D1 carrying N-terminal 7×His and Avi tags and a Prescission protease cleavage site was expressed in *E. coli* BL21 (DE3) and purified using Ni-NTA affinity chromatography as previously described (85,99). Briefly, cells were grown in LB + 0.2% Glucose supplemented with 50 μg/ml Kanamycin to an OD600 ~0.5 and expression was induced with 0.5 mM IPTG at 37 ºC for 4 h. Cell pellets were resuspended in 40 mM sodium phosphate, pH 8, and frozen at -20 ºC until use. Thawed cell pellets were supplemented with DNaseI (80 μg/ml) and Lysosyme (1 mg/ml) and incubated on ice for 30 min, followed by addition of PMSF (1mM) and lysis by sonication or French Press. The lysate was cleared by centrifugation at 13,000 × g for 40 min at 4 ºC and the supernatant filtered (0.45 μm) before loading onto a 20 ml Ni-NTA column at 1-2 ml/min. The column was then washed successively with 120 ml each of buffers 1-3 (Buffer 1: 40 mM Tris·HCl, pH 8, 300 mM NaCl, 1% Triton X-100; Buffer 2: 40 mM Tris·HCl, pH 8, 300 mM NaCl, 25 mM Sodium Cholate; Buffer 3: 40 mM Tris·HCl, pH 8, 300 mM NaCl. The column was then attached to an ÄKTA FPLC for elution using a step imidazole gradient (buffer A: 40 mM Tris·HCl, pH 8, 100 mM NaCl; buffer B 40 mM Tris·HCl, pH 8, 100 mM NaCl, 250 mM imidazole) as follows: 0-18% buffer B over 1 CV, 18% for 4 CV, 18-100% over 30 CV, 100% for 1 CV. MSP containing fractions were pooled, dialyzed against 4 mM Tris·HCl, pH 8, 15 mM NaCl, lyophilized, and stored at -80 ºC until use. Yield is about 20 mg MSP per liter of cells.

Isoform a4 2×FLAG stable expression and growth

Suspension HEK cells (293F, ThermoFisher) were maintained in Freestyle media (Gibco) in vented shake flasks at 37 ºC in a humidified 8% CO_2 atmosphere. Cells (30 ml culture, 1×10^6 viable cells per ml) were transfected with a pcDNA3.1 vector containing the coding sequence for isoform *a*4 (ATP6V0a4) carrying a C-terminal 2×FLAG tag (78) using 293Fectin reagent according to the manufacturer's instructions. Twenty-four hours post transfection, 200 μg/ml Geneticin was added to select for cells containing the Kan/Neo^R marker from the vector. Media and antibiotics were changed every three days and samples periodically taken for analysis of protein expression by western blot. As the culture was only intended for protein purification, a monoclonal population was not selected for. For V-ATPase purification, cells were grown in Freestyle medium in 1.6 l batches, harvested at a density of ~3×10^6 cells/ml by centrifugation at 300 × g for 15 min at 20 ºC (~20 g cell pellet per 1.6 liter growth). The supernatant was discarded and cells immediately flash frozen in liquid nitrogen and stored at -80 ºC until use.

Purification of human V-ATPase in lipid nanodiscs

Frozen cell pellets from 1.6 l culture were weighed (~20 g) and an equal volume TBSE + protease inhibitors (20 mM Tris·HCl, pH 7.2, 150 mM NaCl, 0.5 mM EDTA containing 4 µg/ml Pepstatin, 4 µg/ml Leupeptin, 1 µg/ml Chymostatin and 1 mM PMSF) added and thawed in a room temp water bath. Cells were lysed by 30 strokes with a Dounce homogenizer, centrifuged at 720 × g for 5 min at 4 ºC to pellet unbroken cells and nuclei, and the supernatant was saved on ice. The pellet was resuspended in an equal volume of TBSE + protease inhibitors and the Dounce homogenization was repeated (twenty strokes) to lyse any remaining unbroken cells, followed by another centrifugation under the same conditions. Supernatants were pooled and further clarified by another round of centrifugation as above. The resulting supernatant was centrifuged in a 70 Ti rotor at 14,629 × g for 1 h at 4 ºC and the pellet resuspended in 8 ml of 15 mg/ml MSP in TBSE + protease inhibitors, brought to 1% dodecyl maltoside, and rotated for 45 min at 4 ºC. The MSP to membrane protein ratio is ~1:1. The sample was centrifuged at 16,000 × g for 15 min to remove insoluble material followed by addition of 0.4 g/ml Bio-Beads SM-2 to the supernatant with rotation continued at 4 ºC for 2 h. Bio-Beads were removed by centrifugation at ~2000 × g for 5 m at 4 ºC, and the supernatant was mixed with 1.5 ml FLAG resin (Sigma; pre-equilibrated in TBSE), rotated for 1.5 h at 4 ºC, and then poured into a column and allowed to flow through at ~0.5 ml/min. Unbound material was removed by washing with 50 ml TBSE + inhibitors slowly overnight. Bound protein was slowly eluted in TBSE + inhibitors containing 250 µg/ml 3×FLAG peptide (ApexBio) in 1.5 ml fractions (~20 min incubation between collection of each fraction). Fractions were analyzed by silver stain SDS-PAGE and pertinent fractions pooled (usually fractions 2 and 3), concentrated by ultrafiltration to ~600 µl in a 100 kDa MWCO concentrator (VivaSpin) and applied to an 11 ml 20-50% glycerol gradient containing thirteen 2.5% steps. The gradient was centrifuged in an SW41 rotor at 176,433 × g for 14 h at 4 ºC. Fractions (820 µl) were collected from the top of the gradient, analyzed by western blot and concentration determined using a modified Pierce BCA assay.

Western blot

Samples were separated on gradient SDS-PAGE gels (4-20% acrylamide) and transferred to low fluorescence PVDF membranes in a discontinuous Tris-CAPS buffer system (supplemented with 1% SDS, cathode; 15% methanol, anode) using a TransBlot Turbo (Bio-Rad). Membranes were blocked for 30 min in TBST (20 mM Tris·HCl, pH 7.4, 150 mM NaCl, 0.05% Tween-20) containing 5% non-fat dry milk and then incubated with primary antibodies in TBST containing 1% non-fat dry milk (rabbit anti-A and mouse anti-FLAG; 1:2000 dilution each) at room temp for 2 h. Membranes were washed 3× in TBST and incubated in secondary antibodies in TBST containing 1% non-fat dry milk (Goat anti-rabbit 800CW,

1:10,000; Goat anti-mouse Alexa Fluor 488, 1:2000) for 1 h, washed again 3× in TBST and dried before imaging on a Sapphire Azure scanner with an IR attachment.

Activity measurements

ATPase activity measurements were performed at 37 ºC using a coupled enzyme assay containing 50 mM HEPES, pH 7.5, 25 mM KCl, 0.5 mM NADH, 2 mM phosphoenol pyruvate, 5 mM ATP and 30 U/ml each of pyruvate kinase and lactate dehydrogenase. The rate of decrease in absorbance at 340 nm was converted to the rate of ATP hydrolysis using NADH's extinction coefficient of 6,220 $(M\cdot cm)^{-1}$. Concanamycin A was added to the assay at 200 nM from a 100 µM stock in DMSO.

Transmission electron microscopy

For image analysis, micrographs of negatively stained samples were recorded on a Gatan OneView (4k × 4k) CMOS camera on a JEOL-JEM2100F transmission electron microscope operating at 200 kV. A dataset of ~15,400 particle images collected from 40 micrographs was sorted into 24 classes using reference-free classification and multi-reference alignment protocols as described in (75).

Mass spectrometry sample preparation and analysis

Excised gel bands were subjected to in-gel trypsin digestion. Briefly, gel pieces were washed with 50 mM ammonium bicarbonate in 50% acetonitrile (ACN), reduced with dithiothreitol (DTT) and alkylated with iodoacetamide. Gel pieces were washed again, and impregnated with 75 µl of 5 ng/µl trypsin (Promega, V5280) solution overnight at 37 °C. Peptides were extracted using solutions of 50% and 80% ACN with 0.5% formic acid, and the recovered solution dried in a vacuum concentrator. In-solution digestion was performed using filter-assisted sample preparation (FASP). Briefly, 10 mM Tris pH 8.5, 0.4% (w/w) SDS and 10 mM DTT were added to the sample and disulfides reduced by heating to 95 °C for 5 min. After cooling to room temperature, samples were transferred to a 10 kDa MWCO ultrafiltration vessel and 200 µl of 8 M urea containing 100 mM Tris pH 8.5 (UT) was added before centrifugation to near-dryness. Cysteine residues were alkylated with 50 mM iodoacetamide for 25 min the dark, followed by centrifugation. Retained proteins were washed three times with UT, followed by three washes in 50 mM ammonium bicarbonate. Trypsin was added at a ratio of 1:50 (E:S) in 40 µl of 50 mM ammonium bicarbonate and digestion was allowed to proceed overnight at 37 °C. The resulting peptides were collected by centrifugation, and the filter was washed using 50 µl of 1% trifluoroacetic acid. This wash was added to the collected peptides. The peptides were desalted using mixed-mode cation exchange (MCX) stage tips. The 200 µl tips were packed with two cores of Empore MCX material made using 14 gauge blunt needles. The sorbent was conditioned with ACN, washed with solvent A (3% ACN in water

with 0.2% TFA), up to 10 µg of sample loaded, washed twice with solvent A, washed once with solvent B (65% ACN in water with 0.1% TFA) and eluted using 65% ACN in water containing 5% (v/v) of ammonium hydroxide. The desalted peptides were dried in a speed-vac. For LC-MS/MS, samples were dissolved in water containing 2% ACN and 0.5% formic acid to ~0.25 µg/µl and 2 µl (0.5 µg) injected onto a pulled tip nano-LC column (75 µm inner diameter packed to 25 cm with 2.2 µm C18 particles) for peptide separation using a 3 – 28% ACN gradient over 60 min, followed by a 7 min ramp to 85% ACN. The column was connected inline with an Orbitrap Lumos via a nanoelectrospray source operating at 2.2 kV. The mass spectrometer was operated in data-dependent top speed mode with a cycle time of 2.5 s. MS^1 scans were collected at 60000 resolution with a maximum injection time of 50 ms. HCD fragmentation was used followed by MS^2 scans in the Orbitrap at 15000 resolution with 100 ms maximum injection time. Peak-lists were generated using Spectrum Selector, Proteome Discoverer (version 2.4, Thermo Scientific). The MS data was searched using SequestHT in Proteome Discoverer, v 2.4 against the Human proteome from Uniprot, containing 70709 sequences (2018), concatenated with common laboratory contaminant proteins. Enzyme specificity for trypsin was set to semi-tryptic with up to 2 missed cleavages. Precursor and product ion mass tolerances were 10 ppm and 0.6 Da, respectively. Cysteine carbamidomethylation was set as a fixed modification. Methionine oxidation and protein N-terminal acetylation were set as variable modifications. The output was filtered using the Percolator algorithm with strict FDR set to 0.01. Protein false discovery rate was set to 0.01 using the Protein FDR validator node. Label-free quantification was performed in Proteome Discoverer. Peptide features were detected using the Minora node, allowing only high confidence PSMs. Quantification considered unique and razor peptides and was based on peak abundance. Normalization used total peptide amount and protein abundance was calculated using summed peptide abundances.

Table S1: Key resources

Reagent	Supplier	Catalog Number	Notes
**Recombinant Anti-ATP6V1A (Rabbit)**	Abcam	ab199326	1:2000 WB
**Monoclonal ANTI-FLAG® M2 antibody produced in mouse**	Krackeler	45-F3165-1MG	1:2000 WB
**Goat anti-Mouse IgG (H+L) Highly Cross-Adsorbed Secondary Antibody, Alexa Fluor Plus 488 (Invitrogen)**	Life Technologies Corporation	A32723	1:2000 WB
**Goat anti-Rabbit IgG H&L (IRDye® 800CW) preadsorbed**	Abcam	ab216773	1:10,000 WB
**Freestyle 293-F cells (Gibco)**	Thermofisher	R79007	Cell Culture
**FreeStyle™ 293 Expression Medium Gibco**	Thermofisher	12338026	Cell Culture
**Geneticin™ Selective Antibiotic (G418 Sulfate)**	Thermofisher	10131035	Cell Culture
**Gibco™ 293fectin™ Transfection Reagent**	Thermofisher	12347019	Cell Culture
**Gibco™ Opti-MEM™, Reduced Serum Medium**	Thermofisher	31985062	Cell Culture
**3X FLAG Peptide 25mg**	Apex Bio	A6001	Purification V_1V_o
**ANTI-FLAG® M2 Affinity Gel**	Krackeler	45-A2220-5ML	Purification V_1V_o
**Lactate Dehydrogenase**	Krackeler	45-L2500-10KU	ATPase assay
**Pyruvate Kinase from rabbit muscle Type II**	Krackeler	45-P1506-25KU	ATPase assay
**Nickel NTA Agarose Beads**	GoldBio	H-350-50	Purification MSP
**n-Dodecyl-□-D-Maltopyranoside, Anagrade**	Anatrace	D310 25 GM	Purification V_1V_o
**Bio-Beads SM2 Adsorbent media**	BioRad	1528920	Purification V_1V_o

A

	Isoform	Ubiquitous	Kidney	Bone	Brain
V_1	B1		x		
	B2	x		x	x
	C1	x		x	x
	C2		x		
	E1	x	x	x	x
	E2				
	G1	x		x	
	G2				x
	G3		x		
V_o	a1				x
	a2	x			
	a3			x	
	a4		x		
	d1	x			x
	d2		x	x	
	e1	x		x	
	e2		x		x

C

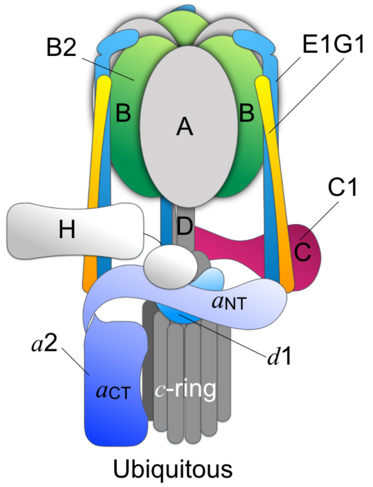

B

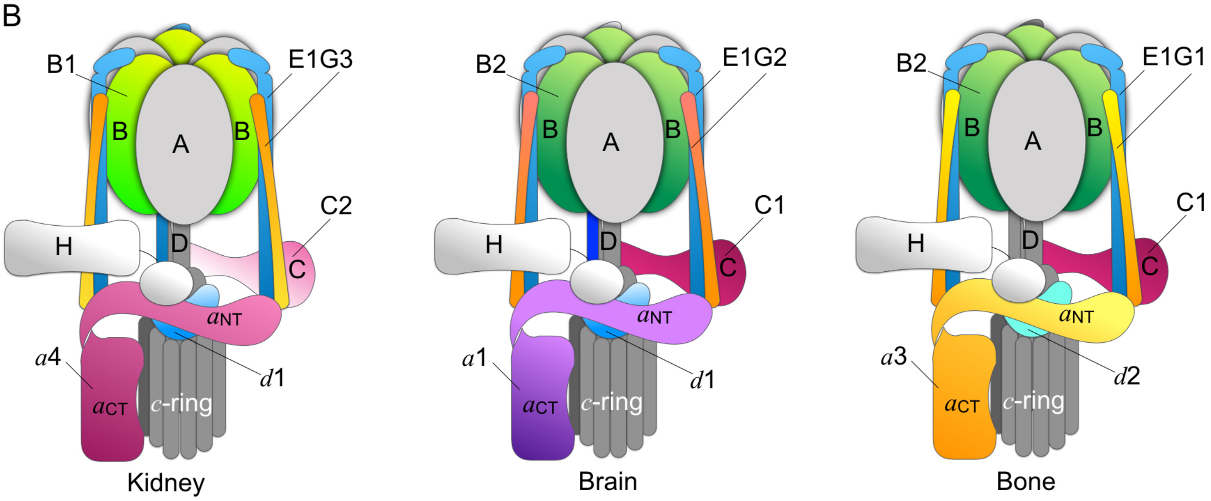

Figure S1: V-ATPase isoforms and specialized complexes

(A) Distribution of expression of ubiquitous (green), tissue enriched (yellow) or restricted (red) isoforms of V_1 and V_o subunits in selected tissues. “Enriched” isoforms are also found in other locations and “restricted” ones have a narrow range of tissues in which they are expressed. Note, the testis-specific isoform, initially termed E1 (89), is currently annotated in Uniprot as E2 and we are using that nomenclature in this manuscript as our mass spectrometry analysis employed the Uniprot database. (B) Schematic representation of specialized isoform containing complexes based on available structural, experimental interaction, and/or tissue expression data (22,24,44,45,66). (C) An example of a presumptive “Ubiquitous” complex.

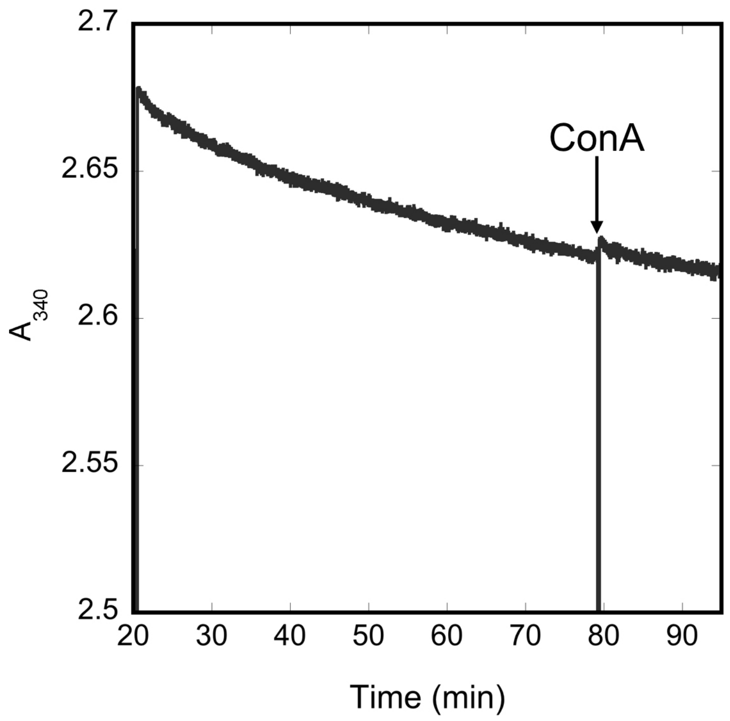

Figure S2: ATPase activity of detergent solubilized *a*4-containing V-ATPase

ATPase activity of detergent solubilized *a*4-containing V-ATPase. The low level of activity (0.003 U/ml) is not sensitive to inhibition by ConA (68% activity remains after treatment), indicating that most of the enzyme is uncoupled.

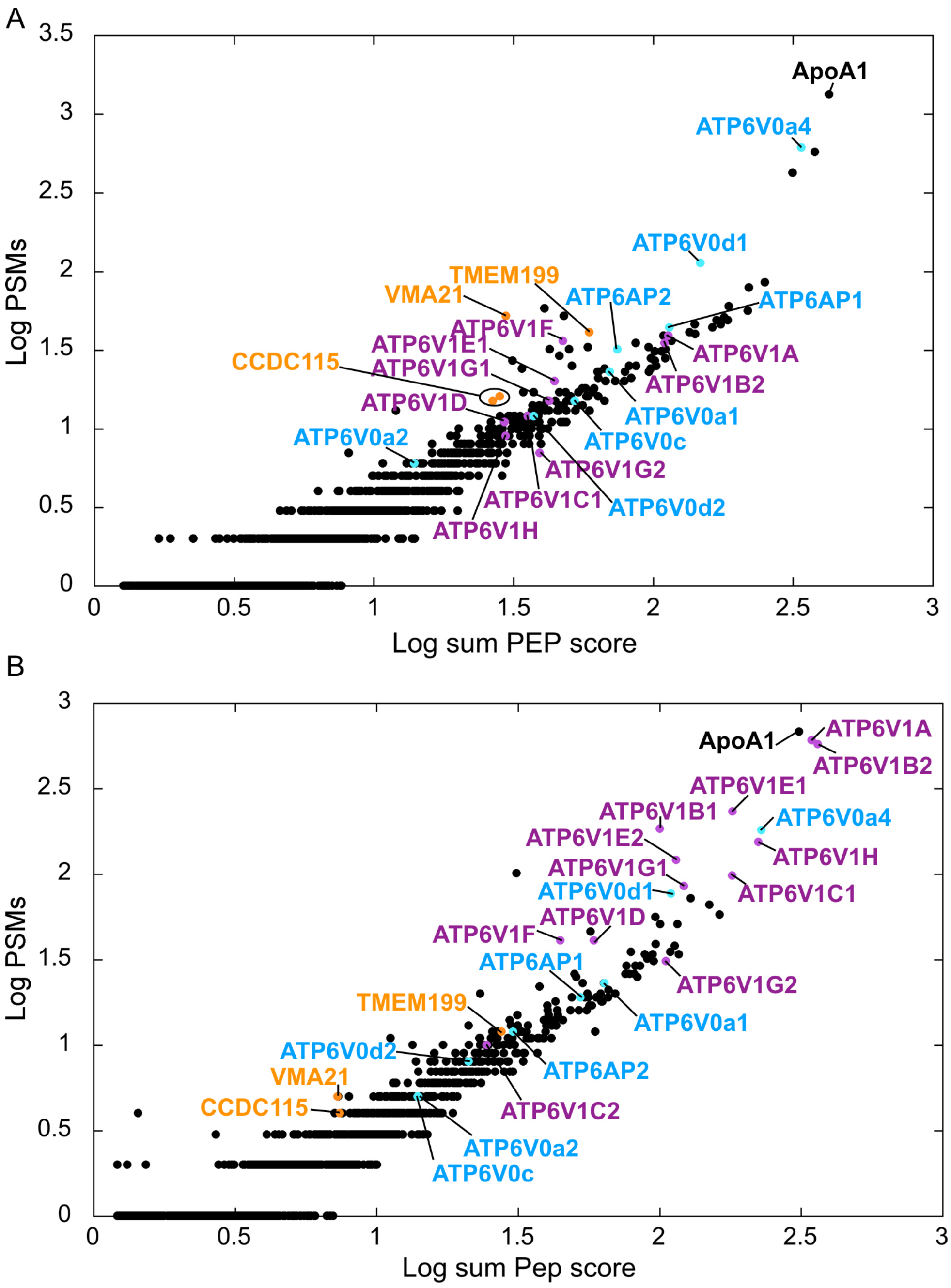

Figure S3: Solution mass spectrometry analysis of additional gradient fractions

Plot showing V-ATPase subunits (gene names are labeled with V_1 subunits *purple*, V_o *blue*) and assembly factors (*orange*) identified in density gradient fractions 5 (A) and 7 (B) (see main text **Fig. 2C, D**). Note that (A) lacks the high scoring V_1 subunits (*purple*) seen in (B), consistent with EM analysis (**Fig. 2F**) showing only V_o in that fraction. The two hits for CCDC115 in (A; *circle*) were identified as two isoforms (accession numbers Q96NT0 and B8ZZ99) but as the associated peptides differ only in a single amino acid, the significance of this is as yet unclear. PSM, peptide spectrum matches; sum PEP score; sum of the negative log of the PEP (posterior error probability) of associated PSMs.

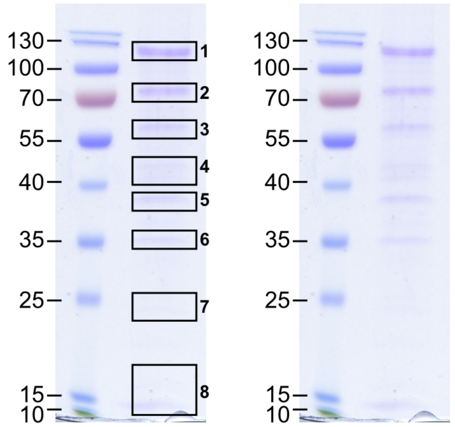

Band no	Accession #	Subunit	Unique Peptides	% Coverage	PSM	Sum PEP Score
1	Q9HBG4	a4	26	28	270	99.773
1	Q9Y487	a2	5	9	18	16.699
1	Q93050	a1	2	5	15	11.75
2	P38606	A	15	22	116	
2	Q9HBG4	a4	2	2	2	
3	P21281	B2	21	35	205	95.949
3	Q9UI12	H	18	34	59	58.984
3	C9JL73	B1	3	14	53	29.09
4	P21283	C1	14	27	61	30.037
4	Q15904	ATP6AP1	5	9	18	7.022
4	F5GYQ1	d1	2	5	4	3.872
4	P21281	B1	2	4	2	3.305
4	Q8NEY4	C2	1	4	3	3.175
5	F5GYQ1	d1	12	23	124	30.509
5	A0A1B0GVW0	ATP6AP2	11	17	38	27.885
5	Q8N8Y2	d2	1	7	2	4.521
6	P36543	E1	12	28	40	
6	Q9Y5K8	D	7	22	14	
7	Q96NT0	CCDC115	4	21	9	
8	Q8N511	TMEM199	3	14	3	
8	O75348	G1	2	8	2	

Figure S4: Mass spectrometry identification of proteins excised from SDS-PAGE

Coomassie blue stained SDS-PAGE gel of pooled peak glycerol gradient fractions (**Fig. 2C,D**). Bands that were excised from the gel for identification by mass spectrometry are boxed and numbered and a table of data from the analysis is shown below the gel. Accession numbers are from Uniprot. The Coomassie stained gel is shown without boxes as well, for clarity. Gel bands were identified from two independent purifications, see below **Fig. S5**.

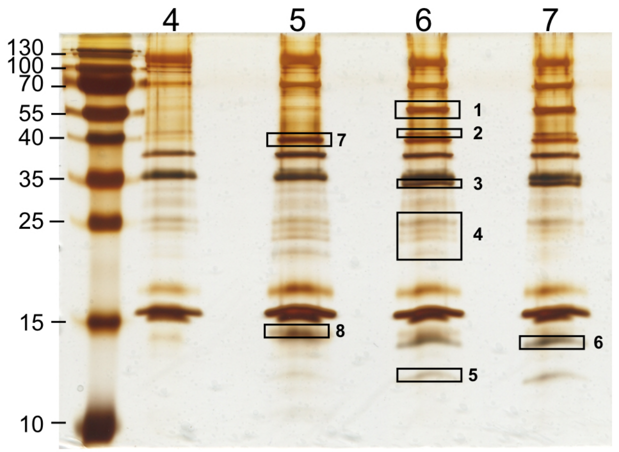

Band no	Accession #	Subunit	Unique Peptides	% Coverage	PSM	Sum PEP Score Score
1	P21281	B2	69	65	623	361.38
1	Q9UI12	H	45	46	154	173.903
1	C9JL73	B1	7	41	159	116.533
2	P21281	B2	6	14	7	
2	P21283	C1	3	7	3	
2	Q15904	S1	2	4	2	
3	P36543	E1	15	46	272	103.166
3	Q9Y5K8	D	16	34	65	55.415
4	Q8N511	TMEM199	13	26	19	27.231
4	Q96NT0	CCDC115	4	32	4	9.631
5	Q16864	F	10	58	34	35.638
5	A0A1B0GTB0	ATP6AP2	4	7	5	9.209
6	O75348	G1	22	73	74	74.253
6	Q3ZAQ7	VMA21	3	22	4	5.436
7	P21283	C1	10	22	13	22.467
7	F5GYQ1	d1	10	17	13	20.693
7	Q15904	ATP6AP1	3	4	5	4.274
8	Q3ZAQ7	VMA21	4	28	12	

Figure S5: Mass spectrometry identification of proteins excised from silver stained SDS-PAGE
Silver stained SDS-PAGE gel showing gradient fractions (fraction numbers indicated above each well). Bands that were excised from the gel for identification by mass spectrometry are boxed and numbered and a table of data from the analysis is shown below the gel. Accession numbers are from Uniprot.

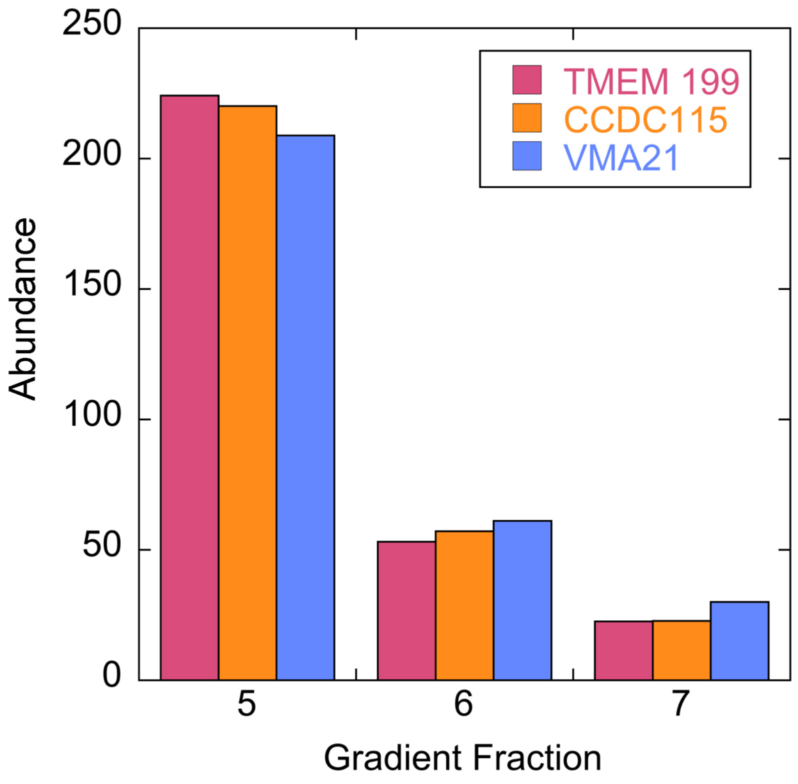

Figure S6: Mass spectrometry label-free quantification of gradient fractions

Relative abundance of assembly factors TMEM199, CCDC115 and VMA21 in three gradient fractions (see main text **Fig. 2C, D**) as determined by label-free quantitation of mass spectrometry data.
